# Central precocious puberty secondary to peripheral precocious puberty due to a pineal germ cell tumor: a case and review of literature

**DOI:** 10.1186/s12902-023-01494-0

**Published:** 2023-10-26

**Authors:** Han Chen, Cai-Yan Mo, Li-yong Zhong

**Affiliations:** Department of Endocrinology, Beijing Tiantan Hospital, Capital Medical University, Beijing, 100070 China

**Keywords:** Precocious puberty, Melatonin, Pineal gland, Germ cell tumor

## Abstract

**Background:**

The pineal lesion affecting melatonin is a rare cause of central precocious puberty by decreasing the inhibition of hypothalamic–pituitary–gonadal axis. Germ cell tumor secreting human chorionic gonadotropin is a rare cause of peripheral puberty.

**Case presentation:**

A 5.8-year-old male presented facial hair and phallic growth, deepened voice, and accelerated growth velocity for 6 months. The elevated human chorionic gonadotropin level with undetectable gonadotropin levels indicated peripheral precocious puberty. Brain imaging revealed a pineal mass and further pathology indicated the diagnosis of teratoma. During chemoradiotherapy with operation, the elevated human chorionic gonadotropin level reduced to normal range, while the levels of gonadotropins and testosterone increased. Subsequently, progressing precocious puberty was arrested with gonadotrophin-releasing hormone analog therapy. Previous cases of transition from peripheral precocious puberty to central precocious puberty were reviewed. The transitions were caused by the suddenly reduced feedback inhibition of sex steroid hormones on gonadotropin releasing hormone and gonadotropins.

**Conclusions:**

For patients with human chorionic gonadotropin-secreting tumors, gonadotropin levels increase prior to sex steroid decrease, seems a sign of melatonin-related central PP related to melatonin.

## Background

 Central nervous system neoplasm is considered as one of the important etiologies of precocious puberty (PP). The neoplasm in proximity to the hypothalamic-pituitary region or intracranial radiotherapy focused on this region [1] may activate the gonadotrophin releasing hormone (GnRH) neuron in advance, causing central PP. Human chorionic gonadotropin (hCG) secreted by tumors may mimic luteinizing hormone (LH) to promote the excessively sex steroid production, causing peripheral PP. In addition, human reproductive cycles are regulated by melatonin, a central gonadal inhibition hormone secreted from the pineal gland [2–4]. The pineal lesion could impact the structure and function of pineal gland, potentially affecting the level of melatonin and then causing central PP [5–13]. Consequently, pineal tumors may present PP via either damging the regulation of pineal and melatonin or mimicking LH funcion by circulating hCG. However, only cases of peripheral PP due to pineal germ cell tumors have been reported to date. The transition from peripheral to central PP is often seen after treatment of the underlying cause. Currently, the loss of feedback inhibition on the hypothalamus and pituitary has been demonstrated as a crucial trigger. Primary gonadal or adrenal diseases which featured elevated sex steroid levels are the most common causes of such loss. In addition, maturation of the HPG axis after prolonged exposure to the elevated sex steroids is believed to be a contributing factor. However, it is unclear whether there are other mechanisms that induce peripheral PP progress to central PP. Herein, we proposed that the pineal hCG-secreting tumor that occurs prior to the expected or normal pubertal onset age may also induce this transition. To further discuss the underlying mechanism, reported cases of central PP secondary to peripheral PP with available data were also reviewed and summarized.

## Case presentation

**Fig. 1:**
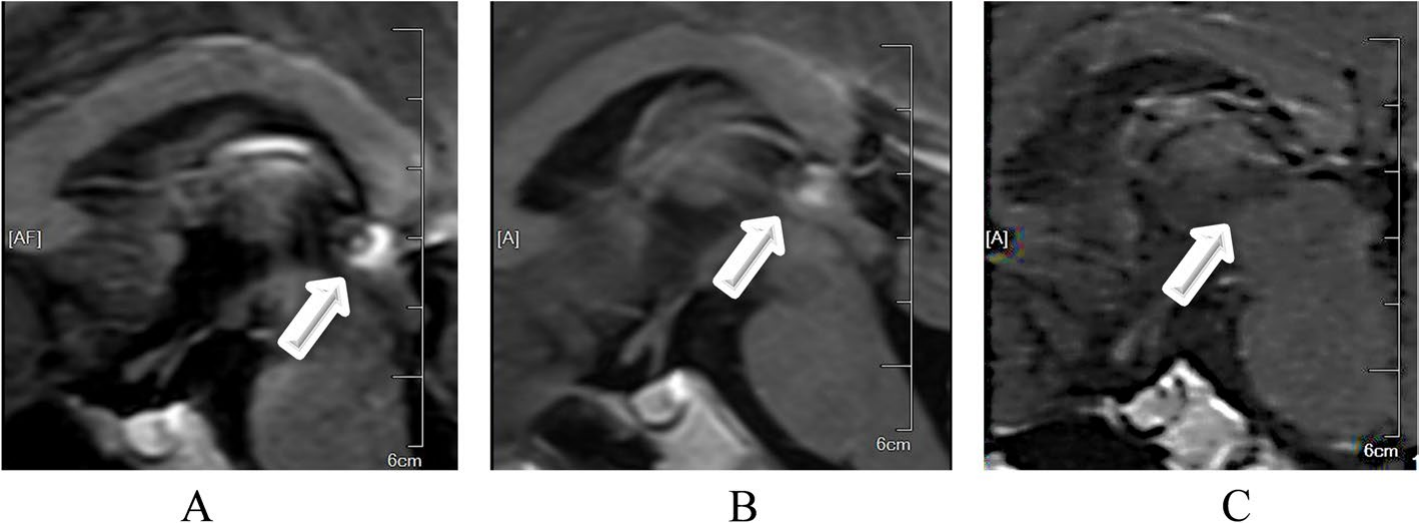
Contrast-enhanced brain MRI. (**A**) Sagittal contrast-enhanced brain MRI at diagnosis, (**B**) sagittal contrast-enhanced brain MRI after 2 courses of chemotherapy, (**C**) sagittal contrast-enhanced brain MRI after chemoradiotherapy with additional neurosurgical operation.1**A** showed a homogenously enhancing pineal mass (marked in white arrow); 1**B** showed the similar tumor imaging (marked in white arrow) to 1**A** although a mild shrinkage; 1**C** showed a disorder of pineal structure (marked in white arrow)

**Fig. 2:**
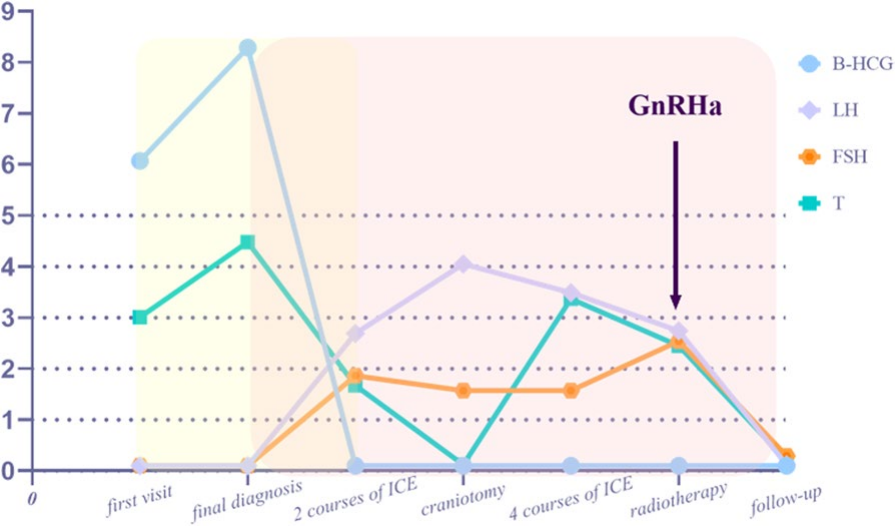
Gonadal hormone profile and hCG concentration. According to the therapeutic strategy, the PP of this patient could be divided into GnRH-independent stage (marked in pink box), GnRH dependent stage (marked in yellow box) and the transient stage (marked in the overlap of two boxes). hCG, human chorionic gonadotropin; LH, luteinizing hormone; FSH, follicle stimulating hormone; T, testosterone; ICE, ifosfamide,carboplatin and etoposide; WRBT, whole brain radiation therapy

A 5.8-year-old boy was admitted to our hospital with a 6-month of facial hair and phallic growth, deepen voice and accelerate growth velocity. The Tanner stage was II for genital development and II-III for pubic hair. Both side testicular volumes were 4 mLby Prader. Family history of PP was denied. The serum sex hormone profile indicated prepubertal levels of gonadotropins with basal LH < 0.1IU/L (prepubertal range < 0.1–0.3 IU/L) and basal FSH < below 0.1 IU/L (prepubertal range < 0.1-3), and pubertal testosterone level (4.48 ng/ml, prepubertal: <0.2-1ng/mL). His height reached 129 cm (+ 2.4 standard deviation scores,) with a high serum insulin like growth factor-1 level (+ 3.5 standard deviation scores) in National-specific charts [14]. Other anterior pituitary hormonal profiles were normal. HCG was detectable both in serum and in cerebrospinal fluid (CSF), 7.12 and 15.66 IU/L separately. Bone age (BA) was advanced to 8 years old. Contrast-enhanced brain magnetic resonance imaging (MRI) revealed a homogeneously enhanced pineal mass (Fig. 1A). The elevated serum hCG level and pineal mass on imaging suggested the clinicial diagnosis of intracranial germ cell tumor (iGCT) and then two courses of ICE chemotherapy (ifosfamide, carboplatin and etoposide) were performed. Repeated MRI did not reflect a significant shrinkage of the pineal lesion (Fig. 1B) although hCG (< 0.1 IU/L, range 0-2.6 IU/L) has been normalized. Meanwhile, basal serum gonadotropin levels (LH 2.96 and FSH 1.86 IU/L, separately) unexpectedly increased (Fig. 2). Considering the possibility of mixed germ cell tumor due to elevated hCG level and poor response to chemotherapy [15, 16], the patient underwent a neurosurgical operation and the histological result revealed a mature teratoma. Subsequently, he continued to receive two additional courses of ICE chemotherapy and the radiotherapy targeting the whole brain (24 Gy) plus the pineal region boosting (12 Gy). During this period, his gonadotropin and testosterone levels were within the normal pubertal range, although with a transient change (Fig. 2). After operation and chemoradiotherapy, MRI indicated the morphological disorders of the pineal gland without an enhanced lesion (Fig. 1C). The patient’s puberty progressed with the Tanner stage III for genital development and stage III for pubic hair. Meanwhile, the patient’s height reached 134 cm (+ 2.7SD) with a bone age of 12 years. To postpone epiphyseal closure and save growth potential, GnRH analogue therapy was started to suppress gonadal activation. This boy decided to return to the local hospital to continue GnRH analogs therapy. Through telephone survey after 2 months of GnRH analogue therapy, the patient’s gonadotropin levels normalized (LH 0.29 and the FSH 0.13 IU/L) and premature presentations gradually regressed. After 18 months of regular GnRH analogue therapy, the patient’s LH and FSH remained undetectable while his precocious puberty regressed. The growth velocity is approximately 5 cm per year.

## Review of literature

We systematically searched PubMed database with “central PP” and “peripheral PP” from inception through July 1st, 2022. No language restrictions were imposed. A hand search through reference lists of relevant primary and review articles was also performed for completeness. Each of the two review authors (HC and C-Y M) independently screened articles for inclusion based on title and abstract and reviewed relevant articles as full text. Disagreement during the review process was resolved by consensus through involvement of a third review author (L-Y Z). A total of 25 original articles involving 51 patients with the transition from peripheral to central PP were reviewed and summarized (16–41) (Table 1). Most of the patients were male (M:F = 11:6), with a CA range of 2–9 years and approximate BA advancement of 1–6 years. All patients had significantly elevated sex steroid levels, with testosterone levels ranging from 196 to 625 ng/dl in males and estradiol levels ranging from 20 to 90 pg/ml in females. Twenty-one patients developed peripheral PP due to congenital adrenal hyperplasia (11 males and 10 females). On admission, CA range and approximate BA advancement range were 3–8 years and 3–16 years, respectively, with symptom intervals ranging from 3 to 14 months. Central PP occurred approximately 6–36 months after the replacement of glucocorticoid and mineralocorticoid for congenital adrenal hyperplasia. Twelve patients developed peripheral PP due to adrenal tumor (7 males and 5 females). On admission, CA range and approximate BA advancement range were 4.5-8 years and 1.5-6 years, respectively, with symptom intervals ranging from 3 to 36 months. Central PP occurred approximately 2–6 months after adrenalectomy for adrenal tumor. Ten patients developed peripheral PP due to the gonadal tumors, including 8 males with Leydig cell tumors and two females with ovary granulosa cell tumors. On admission, CA range and approximate BA advancement range were 5–9 years and 2–6 years, respectively, with the symptom intervals ranging from 6 to 36 months. Central PP occurred approximately 3–18 months after the surgery for tumors. Five male patients developed peripheral PP due to familial male-limited PP. On admission, CA range and approximate BA advancement range were 2–7 years and 3–5 years, respectively. One patient had a symptom interval of 36 months, while the others’ were unclear. Antiandrogen drugs were supplied. Central PP occurred 18 months after antiandrogen treatment in one patient and the duration from peripheral PP to central PP of other patients were unclear. The other patients developed peripheral PP due to McCune-Albright and then progressed to central PP without further details. Only one 6-years old boy with pineal teratoma development peripheral PP and developed central PP at 8 years old, 8 months after his surgery for tumors. Except one male patient received cyproterone acetate, all patients arrested their central PP by GnRH analogues.

**Table 1:** Previous cases of peripheral precocious puberty triggered central precocious puberty

Author	Year	Patient (n,gender)	Peripheral precocious puberty							Duration^a^	Central precocious puberty			
			SI (months)	CA (years)	BA advancement (years)	Sex steroid T(ng/ml) E (pg/ml)	Etiology	Therapy	Outcome		CA	BA	Sex steroid hormone	Therapy
Pescovitz OH	1984	3 M, 1 F	N/A	4.88 ± 0.74	N/A	N/A	CAH	hydrocortisone and fludrocortisone	N/A	2.12 ± 1.38 year b	6.75 ± 0.83	10.5–13.5	T 274 ± 60 E 40 ± 15	LHRHa
Criscuolo T	1985	1 M	12	6	6.00	T 270–360	Leydig cell tumor	cyproterone acetate→surgery	regress	6 months after surgery	7.50	N/A	T 280	cyproterone acetate
Pescovitz OH	1985	1 F	36	4.5	6.50	T 322	Adrenal tumor	adrenalectomy	regress	4 months after surgery	4.80	12.00	E 32.0	LHRHa
Schmidt H	1998	1 F	N/A	N/A	N/A	N/A	McCune-Albright syndrome	N/A	N/A	N/A	6	11	E 15.6	GnRHa
Ghazi AA	2001	1 M	11	6.5	6.50	T 887	Leydig cell tumor	orchidectomy	regress	3 months after surgery	6.8	N/A	T 141.3	medroxyprogesterone, cyproterone acetate → LHRH
Bajpai A	2006	1 M	3	2.5	5.90	T 224	CAH	hydrocortisone and spironolactone	N/A	3 yr	5.2	15	N/A	GnRHa
Jeha GS	2006	1 M	36	4.5	5.50	T 254	FMPP	testolactone, spironolactone, flutamide	progress	18months	6	13	T 225	GnRHa(Lupron Depot)
Almeida MQ	2008	4 M	N/A	4.13 ± 2.21	3.25 ± 1.47	T 196–266	FMPP	cyproterone acetate or ketoconazole	N/A	N/A	N/A	N/A	N/A	GnRHa
Kiepe D	2008	1 M	24	6.7	5.30	T 312	Leydig cell tumor	surgery	regress	1 year	7.7	13.5	T 47	GnRHa
Bas F	2009	1 F	24	2.5	N/A	E 90	Overy granulosa cell tumor	surgery	progress	7 months after surgery	3.1	7	E 30	GnRHa
Miyoshi Y	2009	1 M	18	6.5	5	T 218	Adrenal tumor	surgery	regress	2 months after the operation	6.80	N/A	T 92	GnRHa
Lignitz S	2011	1 M	12	7	5.50	T 144	Leydig cell tumor	surgery	regress	2 months after the operation	7.2 ^b^	N/A	T 201	GnRHa
Olivier P	2012	1 M	36	9	4.50	T 268	Leydig cell tumor	surgery	testosterone decreased	1 month after surgery	9.1^b^	N/A	T 37	N/A
Santos-Silva R	2013	1 M	12	8	4.00	T 148	Leydig cell tumor	surgery	progress	2 months after surgery	8.2^b^	N/A	T 245	GnRHa
Güven A,	2015	5 M, 5 F	N/A	6.18 ± 2.1	4.53 ± 1.92	T 1.21–625 E2 20-40.1	CAH	hydrocortisone, mineralocorticoid	N/A	0.82 ± 0.54 years	6.78 ± 1.8	11.2 ± 1.7	N/A	GnRHa
Kim MS	2015	1 M	3	8	1.50	T 382	Adrenal tumor	surgery	regress	2 months after the operation	8.2	N/A	T 334	GnRHa
Verrotti A	2015	2 M	4,6	7.5,7.7	2.3,3.3	T 158,	Leydig cell tumor	surgery	regress	4 months after surgery	7.8-8.0	12.5, 12	T 178, 210	GnRHa
Sahana PK	2016	1 M	5.4	N/A	N/A	N/A	CAH	hydrocortisone	N/A	N/A	6.5	12	T 910	LHRHa
Ersoy B	2017	1 F	4	4	5.00	E 34.6 T 67.22	Adrenal tumor	surgery	progress	24 months after surgery	6	10.50	E2 20	GnRHa
Dayal D	2018	4 F, 1 M	14	3.6 ± 0.74	3.2 ± 1.3	N/A	CAH	hydrocortisone and fludrocortisone	N/A	N/A	4.7 ± 1.2	N/A	N/A	GnRHa
Goyal A	2019	1 M	8	7	6.00	T 1190	Adrenal tumor	surgery	regress	6 months after surgery	7.5^b^	N/A	T 72.9	GnRHa
Stecchini MF	2019	4 F, 3 M	6 ≥ 48 months 6 < 48 months	4 ≥ 48 months 3 < 48 months	5 Advanced 1 Not advanced	T 381	Adrenal tumor	surgery combined with chemotherapy or radiotherapy	N/A	N/A	N/A	N/A	N/A	GnRHa(2 F, 1 M)
Singhania P	2022	1 M	4–6	6	6.00	T 293	Leydig cell tumor	surgery	regress	4 weeks after surgery	6.1^b^	N/A	T 28.4	GnRHa
Gaikwad PM	2022	1 F	30	5.5	N/A	E 22.1	Overy granulosa cell tumor	surgery and chemotherapy	N/A	18 months after surgery	7	11	E 11	GnRHa
Cattoni A	2022	1 M	4	6.2	N/A	6.85	mature teratomatous and germinoma	bicalutamide and anastrozole →surgery→chemotherapy and radiotherap	progress	8 months after surgery	8.00	N/A	N/A	GnRHa

*SI* Symptoms intervals; *CA* Chronological age; *BA* Bone age; Patient: *M* Male; *F* Female; *T* Testosterone (1ng/ml = 0.288184nmol/l); *E* Estradiol; *CAH* Congenital adrenal hyperplasia; *FMPP* Familial male-limited precocious puberty; *GnRHa* Gonadotropin releasing hormone agonist; *LHRHa* Luteinizing hormone releasing hormone analogue; *N/A* Not available

^a^The timespan from the peripheral precocious puberty to central precocious puberty

^b^ The timespan was assessed through the chronological age onset of peripheral precocious puberty and of central precocious puberty

## Discussion

Transition from peripheral PP to central PP is rare, which means activating GnRH or inhibiting gonadotropin inhibitory hormone after a period of overexposure to steroids. This transition has been reported in patients with Leydig cell tumors [17–24], adrenal tumors [25–31], congenital adrenal hyperplasia [32–35], overy granulosa cell tumors [36–38], familial male-limited PP [39, 40], McCune-Albright syndrome [41] and pineal mature teratoma [42], probably due to the circulating sex steroid hormones’ suddenly reduced feedback inhibition on gonadotropin releasing hormone and gonadotropins. In addition to primary gonadal diseases, only one case of central PP secondary to extragonadal hCG-secreting iGCT has been reported, and this patient has long-term exposure to endogenous testosterone [42].

**Fig. 3:**
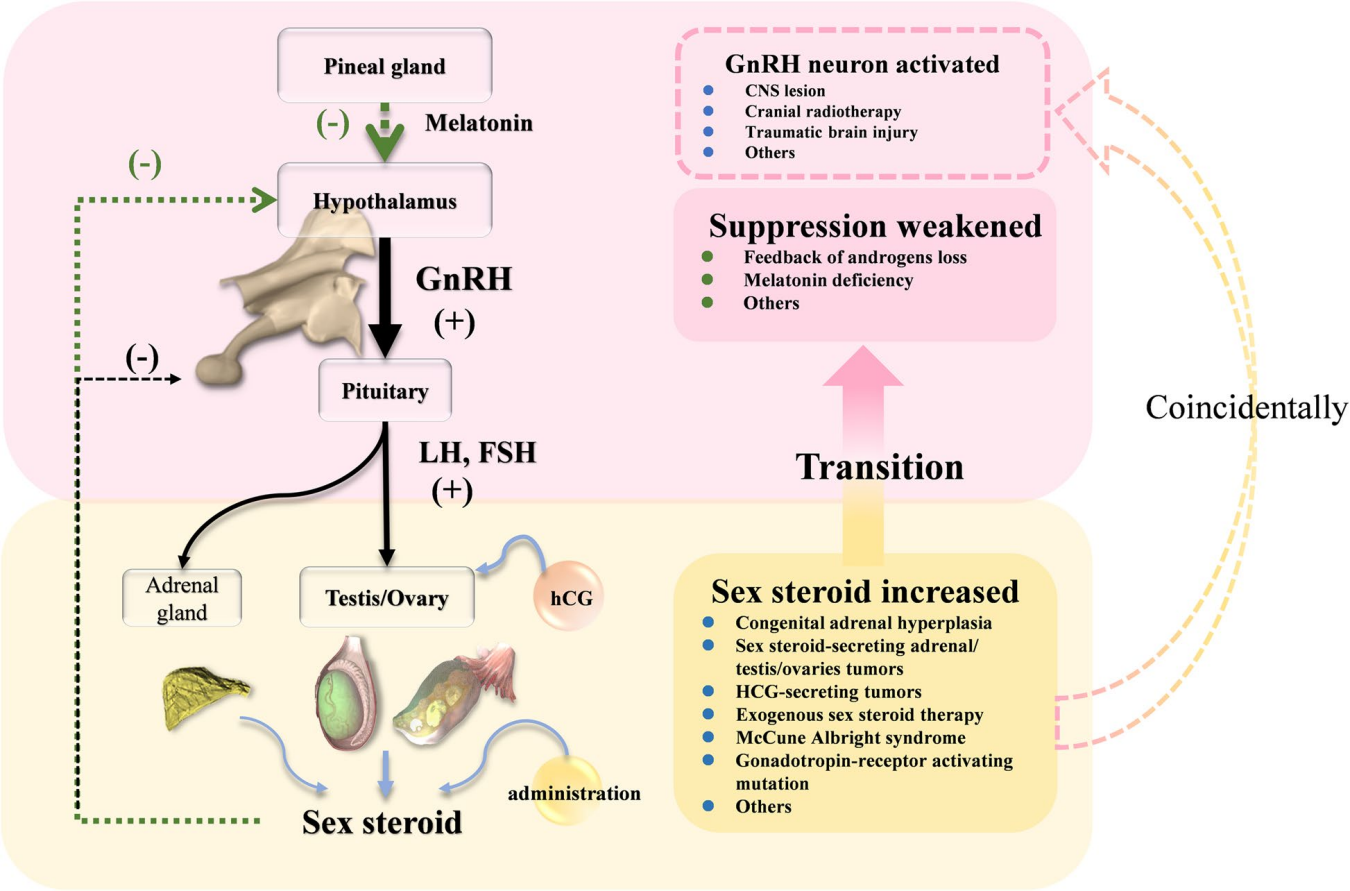
Probably mechanisms of the transition from peripheral to central precocious puberty

In the present study, we describe a young boy with a pineal germ cell tumor and peripheral PP who later developed central PP. Initial peripheral PP of the patient due to serum hCG, which binds to the LH receptor and then acts on the testicular Leydig cell to excessively promote testosterone synthesis. The elevated hCG level in serum and CSF inidicated the diagnosis of iGCT. The residual in pineal lesion after induction chemotherapy indicated the diagnosis of mixed iGCT and following histological results indicated the teratoma component. During the initial 2 courses of chemotherapy, although the hCG level returned to normal range, the patient’s LH and FSH levels increased synchronously with the stably elevated testosterone. Compared to previous cases where patients’ central PP commonly developed after months or even years of peripheral PP regression, the abnormal change in gonadotropin and sex steroid hormone of this patient suggested a probable mechanism different from loss of negative feedback to the gonadotropins (Fig. 3). In our opinion, the transition from peripheral PP to central PP in the present study is likely associated with the destruction of the pineal gland and the impairment of melatonin function (1). Patient’s gonadotropin levels began to increase following the chemotherapy in parallel with a decline of hCG and sex steroid level. The change in the level of circulating sex steroids seems insufficient to regulate hythalamic inhibition during this short peroid (2). The patient received surgery and multidrug therapy for tumor at the age of 5. During this period, the inhibitory role of melatonin in gonadotropin release was usually strong and crucial [43]. The annual rhythm of melatonin during this period may act as a regulator of pubertal onset (3). Most of the published cases of transition from peripheral PP to central PP are adrenal or gonadal diseases, rather than extragonadal diseases. Nevertheless, before central PP activates, adrenal or gonadal diseases manifested long-term and high-level exposure to sex steroid hormone. But for extragonadal diseases, the exposure time is commonly shorter, and level lower. Besides, there was only one case of pineal teratoma that has been reported to developed central PP after peripheral PP. The differences between previous case and oue case are also significant. Firstly, the peak elevated testosterone level in our case seems not high enough to play a crucial role in the hypothalamic threshold. That may be why patients with transition from peripheral to central precocious puberty had adrenal or gonadal disease, or other disease secreting sufficient sex steroid hormone. Additionally, our case developed central precocious puberty immediately after normalizing the hCG, while previous cases always had a time span from peripheral to central precocious puberty. Finally, the symptom interval of the present patient was only six months so that the exposure time is not a long-standing period [4]. Other causes triggering GnRH-dependent puberty, like radiotherapy, co-existence of other lesions in hypothalamus-pituitary region, injury or infection of the central nervous system have been carefully excluded. However, all these presumptions are based on the strong evidence of physiological and the neuroendocrine functions of the pineal gland and melatonin, related to the clinical manifestations and the changes in sex hormones. At present, the clinical assessment of the role of melatonin in precocious puberty remains difficult and thus there is very little data on melatonin levels in peripheral or in the CNS. The difficulties are as follows. First, melatonin samples should be collected under dim light conditions throughout the night after controlling potential risk factors for oxidative stress and inflammation. It seems difficult for malignancy children to collect melatonin sample during their crucial anti-cancer therapies. Next, the reference range of melatonin matched for gender, age, and ethnicity in healthy individuals remain controversial so that interpretation of results may not be straightforward. Finally, in future research, the evaluation in puberty of patients with pineal hCG-secreting tumors should be highlighted, although the hCG-induced PP of most cases would spontaneously regress together with tumor elimination. If possible, collecting the melatonin level at baseline and after therapy should be recommended.

## Conclusion

Clinicians caring for young patients with pineal hCG-secreting tumors should be alert to the secondary central PP, although most hCG-induced peripheral PP would regress after the tumor was eliminated. Gonadotropin levels increasing prior to sex steroid suppression may be a a sign of melatonin-related central PP. It is highly recommended to assess the function of hypothalamic–pituitary–gonadal axis by testing hormones during the treatment period of pineal hCG-secreting tumors.

## Data Availability

The datasets used and/or analyzed during the current study are available from the corresponding author on reasonable request.

## Abbreviations

PP: Precocious puberty
GnRH: Gonadotrophin releasing hormone
hCG: Human chorionic gonadotropin
LH: Luteinizing hormone
FSH: Follicle stimulating hormone
CSF: Cerebrospinal fluid
iGCT: Intracranial germ cell tumor
BA: Bone age
MRI: Magnetic resonance imaging
CA: Chronological age
